# Photosynthetic Traits, Growth, and Yield of Cassava Plantlets From Different Sources and Cultivars

**DOI:** 10.1155/sci5/5512707

**Published:** 2025-10-30

**Authors:** Sovannara Moun, Supawadee Kaewrahun, Anon Janket

**Affiliations:** ^1^Department of Agronomy, Ubon Ratchathani University, Ubon Ratchathani, Thailand; ^2^Department of Agriculture, National University of Battambang, Battambang, Cambodia

## Abstract

Cassava cultivation faces increasing challenges from diseases, particularly cassava mosaic virus. Efficient propagation systems are essential for producing disease-free plants and sustaining production. However, information on the agronomic, physiological, growth traits, and yield of cassava plantlets produced through rapid propagation remains limited. This study aimed to investigate the photosynthetic and agronomic traits, as well as the growth and yield, of cassava plantlets derived from an aeroponic system under both pot and field conditions, using a 3 × 4 factorial arrangement in a randomized complete block design (RCBD). Three cassava varieties—Kasetsart 50 (KU50), Rayong 9 (RY9), and Huay Bong 60 (HB60)—were assigned as Factor A, while four plantlet sources—three derived from aeroponic systems (leaf bud cuttings, mini-cuttings, and normal cuttings) and one from conventional cuttings—were assigned as Factor B. The results indicated that HB60 and RY9 exhibited superior growth, stem diameter, photosynthesis rate, stomatal conductance, transpiration, and higher yields. Notably, RY9 also showed greater plant height and stem diameter, contributing to a higher multiplication rate. Plantlet sources did not significantly affect photosynthetic traits under either pot or field conditions but did increase canopy height, starch yield in the field, and starch content in the pot. Interestingly, the performance of leaf bud cuttings, mini-cuttings, and normal cuttings was comparable to conventional planting methods in terms of photosynthetic traits, yield traits, and harvest index. These findings suggest similarities among cassava cultivars in their responses to different plantlet sources and highlight the potential value of plantlet sources as a consideration for plant propagation programs.

## 1. Introduction

Cassava (*Manihot esculenta* Crantz) is a major root and tuber crop, experiencing an 88% increase in global production over the past 22 years. As a vital food source, cassava is extensively cultivated in tropical and subtropical regions, supporting millions worldwide [1]. In 2022, the leading cassava producers included Nigeria, the Democratic Republic of Congo, Thailand, Ghana, Cambodia, Brazil, Indonesia, Vietnam, Angola, and Mozambique, respectively [2]. Cassava serves multiple purposes, such as direct consumption, starch production, animal feed, and bioethanol production [3]. However, the expansion of cultivation and high demand for planting materials has heightened the risk of regional virus transmission, including Sri Lankan cassava mosaic disease (SLCMD) in Southeast Asia [4].

The severity of cassava diseases is influenced by factors, such as plant age, cultivar, virus strain, and climate. SLCMD can reduce root yields by 16%–33% when propagated from infected cuttings [5]. Although breeding programs in Asia have developed SLCMD-resistant cultivars, the availability of planting materials remains insufficient for farmers. This situation underlines the importance of rapid propagation techniques to produce disease-free planting materials, currently the primary method for controlling disease spread [4–6].

Traditionally, farmers use cassava stems aged 8–12 months for replanting, cutting them into 20–30 cm segments with 6–8 nodes. However, this practice has a low multiplication rate of 1:10 [7]. To address this limitation, researchers have developed rapid multiplication techniques, including tissue culture, the leaf bud technique, mini-cuttings, aeroponic systems, and semihydroponic systems [8–12]. While these methods improve the quality of planting materials, a few studies have evaluated the agronomic performance, photosynthetic traits, and tuber yields of plantlets derived from these techniques. Neves et al. [13] found that cassava plantlets propagated from leaf buds showed reduced agronomic performance, such as shoot weight, branching, stem height, root weight, and root number, compared to those from traditional stem cuttings. However, plantlets derived from leaf buds on the upper part of the plant showed a greater number of branches, while those from both the upper and middle parts had dry matter comparable to traditional cuttings. Field studies of two-node cassava cuttings demonstrated that factors like cutting size, mother plant variety, nodal age, plant age, and fertilization significantly affect shoot production. Notably, cuttings from 7-month-old plants had the highest shoot growth and internode numbers, highlighting the importance of plant age in propagation [14].

Research comparing mini-cuttings with normal cuttings found no significant differences in vegetative growth or starch content, although normal cuttings outperformed mini-cuttings in branching and tuber yield for certain varieties. This suggests that mini-cuttings can be a cost-effective, space-efficient alternative without substantially compromising yield [15]. Additionally, cassava plantlets derived from rapid propagation methods show varying field performance compared to traditional stem cuttings, with genetic diversity influencing branching patterns and tuber yields [16]. Moun et al. [17] reported that combining propagation methods with aeroponic systems increased cassava plantlet production and improved survival rates for shoot cuttings from leaf buds and mini-cuttings.

However, the performance of aeroponically propagated cassava plantlets under natural environmental conditions remains underexplored. There is a notable gap in research comparing aeroponically propagated plantlets with those derived from conventional stem cuttings, particularly in terms of photosynthetic traits, growth, and yield. Environmental factors such as nutrient availability, soil properties, and water access significantly affect cassava growth and yield [18]. Addressing this research gap would provide valuable insights into the adaptability of aeroponically propagated cassava plantlets in natural environments. Furthermore, it would enable comparisons between aeroponic and conventional propagation methods regarding physiological and agronomic traits as well as yield performance. Therefore, this study aims to investigate the photosynthetic traits, agronomic performance, growth, and yield of cassava plantlets derived from aeroponic systems across various cultivars under both pot and field conditions.

## 2. Materials and Methods

### 2.1. Study Area

The study was conducted at the experimental farm and central laboratory of Ubon Ratchathani University, located in Northeast Thailand (15°11′N, 104°53′E, 130 m above sea level). The research comprised both pot and field experiments, carried out between September 2023 and April 2024. During this period, the average temperature ranged from 28.6°C to 32.5°C, with relative humidity between 72.3% and 87.0%. Both experiments were conducted on Roi-et series soil, which is widely distributed across Northeast Thailand.

For the pot experiment, soil samples were collected during soil preparation after sieving and before adding soil to the pots, whereas in the field experiment, soil samples were randomly collected from 6 points before planting at depths of 0–30 and 30–60 cm. The samples were air-dried, sieved, and analyzed for physicochemical properties, including soil texture, pH, organic matter, total nitrogen, available phosphorus, exchangeable potassium, calcium, magnesium, electrical conductivity (EC), and cation exchange capacity (CEC).

The soil used in both experiments was classified as loamy sand, comprising 74.95%–77.87% sand, 19.19%–20.95% silt, and 2.94%–4.29% clay. In the pot experiment, the soil had a pH of 5.65 and an EC of 0.02 ds m^−1^. Organic matter content was 0.57%, total nitrogen was 0.04%, and available phosphorus was 100.63 mg kg^−1^. Exchangeable potassium, calcium, and magnesium were 24.16, 113.71, and 29.39 mg kg^−1^, respectively. The CEC was 10.70 cmol kg^−1^ (Table 1).

In the field experiment, the soil pH ranged from 4.98 to 5.04 at both 0–30 cm and 30–60 depths, with an EC of 0.01 ds m^−1^. Organic matter content ranged from 0.25% to 0.48%, total nitrogen from 0.01% to 0.02%, and available phosphorus from 5.13 to 13.75 mg kg^−1^. Exchangeable potassium ranged from 6.69 to 6.84 mg kg^−1^, calcium from 10.63 to 22.99 mg kg^−1^, and magnesium from 4.60 to 9.31 mg kg^−1^. The CEC ranged from 4.20 to 5.00 cmol kg^−1^ (Table 1).

Overall, the soil used in the pot experiment was more fertile than that of the field experiment. The soil in the field experiment exhibited slightly higher acidity and lower levels of organic matter, total nitrogen, available phosphorus, exchangeable potassium, calcium, and magnesium.

### 2.2. Planting Material Sources

The cassava plantlets used in this study were obtained from previous research conducted by Moun et al. [17]. This earlier research involved a two-phase process to produce a sufficient number of plantlets for subsequent experiments. In the first phase, plantlets were generated from stem cuttings using three methods: leaf bud cuttings, mini-cuttings, and normal cuttings. In the second phase, the plantlets were transferred to an aeroponic propagation system to facilitate rapid multiplication. These plantlets were then utilized in the present study to assess their performance under both pot and field conditions.

### 2.3. Treatments and Experimental Design

A 3 × 4 factorial experiment in a randomized complete block design (RCBD) was employed under both pot and field conditions. The factors included three cassava varieties—Kasetsart 50 (KU50), Rayong 9 (RY9), and Huay Bong 60 (HB60)—assigned as Factor A, and four plantlet sources derived from aeroponic systems (leaf bud cuttings, mini-cuttings, normal cuttings) and conventional cuttings, assigned as Factor B.

The pot experiment consisted of four replications, with two plants per experimental unit. Each pot measured 0.8 m in diameter and 0.5 m in height, containing 340 kg of dried soil. The field experiment had three replications. Before planting for the field experiment, the field was prepared with two rounds of plowing using a 3-disk and a 7-disk tractor, followed by harrowing. Soil ridges were formed with 1 m spacing between adjacent ridges. Each plot measured 5.6 × 3 m (16.8 m^2^) and included three rows, with a spacing of 1 m between rows and 0.8 m between plants within a row, for a total of 21 plants per plot.

For both pot and field experiments, limestone was applied at a rate of 625 kg ha^−1^ to improve soil quality. Organic fertilizer pellets (Bat Cave, TPI brand, Thailand) were applied at the recommended rate of 2100 kg ha^−1^. Chemical fertilizers with a formula of 13-13-21 (N-P_2_O_5_-K_2_O) were applied at a rate of 315 kg ha^−1^ twice, at 1 and 3 months after planting (MAP), following the guidelines of the Ministry of Agriculture and Cooperatives [19]. Foliar fertilizers (20-5-5) were sprayed twice, at 1 and 2 MAP, based on recommendations from the Alliance of Bioversity International and CIAT [20].

Conventional stem cuttings (20 cm) from 10-month-old mother plants served as control treatments. These cuttings were soaked in a thiamethoxam 25% solution (4 g 20 L^−1^ water) for 10 min before planting to prevent insect infestations. Aeroponically derived plantlets (leaf bud cuttings, mini-cuttings, and normal cuttings) were hardened under 50% shade for 1 week before being transplanted into pots or field plots. Planting was done by hoeing to a depth of approximately 30 cm in the center of the pot or along the ridges in the field.

Soil moisture was monitored using tensiometers installed at depths of 30 and 60 cm in both experiments. Drip irrigation was initiated when soil water tension at the 30 cm depth reached −30 kPa and stopped when tension at the 60 cm depth reached 0 kPa, ensuring optimal moisture levels and preventing crop water stress. Hand weeding and insecticide applications were performed as needed to manage weeds and pests.

### 2.4. Data Collection

Plant height, canopy height, internode length, total leaf number, and branching number were measured monthly for each replication, using two plants per replication for the pot experiment and five plants per replication for the field experiment. Measurements began at 4 MAP and continued until 8 MAP. Plant height (cm) was determined from the soil level at the main stem to the uppermost fully expanded leaf. Canopy height (cm) was measured from the node bearing the lowest green leaf to the shoot apex. Average internode length was calculated by dividing the plant height by the total number of nodes. The total number of green leaves and branches was also recorded.

At 4 and 6 MAP, leaf gas exchange parameters including net photosynthesis (Pn), stomatal conductance (Gs), transpiration rate (Tr), and intercellular CO_2_ concentration (Ci) were measured from two plants per replication between 9:00 and 11:00 a.m. on clear-sky days using an LCi-SD photosynthesis system (LCi-SD Ultra Compact Photosynthesis System, ADC BioScientific Ltd, Hoddesdon, UK). These parameters were measured on the central lobe of a fully expanded leaf positioned as the sixth leaf from the shoot tip on the main stem. The maximum quantum yield efficiency of Photosystem II in the light (Fv′/Fm′) was also measured between 9:00 and 11:00 a.m. on the same plants and leaves using a Handy PEA chlorophyll fluorimeter (Handy PEA, Hansatech Instruments Ltd, Lynn, UK). Transpiration efficiency (TE) was calculated as the ratio of Pn to Tr.

At the final harvest, two pots per experimental unit (pot experiment) and five bordered plants per field plot (field experiment) were sampled. Plants were cut at the soil surface and separated into leaves, stems, petioles, and tubers. Fully mature main stems (≥ 15 cm diameter) were measured at the midpoint using a digital caliper (HERO No: HC2215) to calculate the multiplication ratio. Tubers were washed to remove soil, and those exceeding 2 cm in diameter were counted and weighed fresh. A tuber sample (5 kg experimental unit^−1^) was cut into 5-cm segments and analyzed for starch content using a Reimann scale balance.

The fresh weight of each plant part was recorded using a digital scale. Subsamples (at least 10% of each plant part) were collected for further analysis. Leaf samples were used to measure leaf area with a leaf area meter (Licor 3100, Li-Cor Inc., Lincoln, NE, USA). These subsamples were then oven-dried at 72°C for 72 h, or until a constant weight was achieved, and their dry weights were recorded. Total biomass was calculated as the sum of the dry weights of leaves, stems, petioles, and tubers. Total biomass and tuber weights were expressed as kilograms plant^−1^ for the pot experiment and tons ha^−1^ for the field experiment. Starch yield was determined by multiplying starch content by tuber fresh weight. The harvest index (HI) was calculated as the ratio of tuber dry weight to total biomass. Specific leaf area (SLA) was determined as the ratio of leaf area to leaf dry weight.

### 2.5. Statistical Analysis

Analysis of variance (ANOVA) for all parameters was performed according to a factorial arrangement in a RCBD using Statistix Version 10 [21]. Tukey's honest significant difference (HSD) test was applied to compare means at a 95% confidence level.

## 3. Results

### 3.1. ANOVA

The results of the ANOVA showed significant differences among cassava plantlet sources and cassava cultivars for most measured parameters across all sampling times (data not shown). However, there was no significant interaction between cassava plantlet sources and cassava cultivars for most measured parameters across all sampling times. This finding suggests that the cassava cultivars exhibited similar responses to the plantlet sources, with variations in magnitude but consistent overall trends. Consequently, the means are presented only for the main effects (plantlet sources and cassava cultivars).

### 3.2. Growth and Development of Cassava Plantlets in a Pot Environment

**Figure 1:**
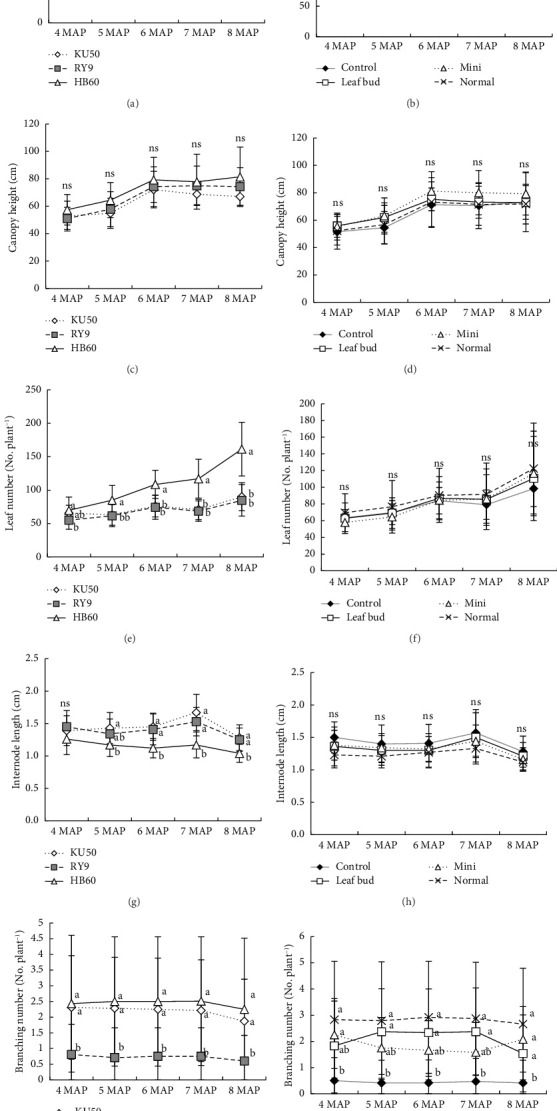
(a) & (b) plant height, (c) & (d) canopy height, (e) & (f) total leaf number, (g) & (h) internode length, and (i) & (j) branching number of three cassava cultivars and in four sources of plantlets under pot experiment conditions. There was no significant interaction between cassava plantlet sources and cassava cultivars for all parameters. Different lowercase letters represent significant differences among cassava cultivars and cassava plantlets at *p-*value ≤ 0.05.

The agronomic development of cassava plantlets from 4 to 8 MAP is presented in Figure 1. Plant height did not show any statistically significant differences across different cassava cultivars and plantlet sources at any of the sampling times (Figures 1(a), 1(b)). Canopy height exhibited minimal variation across the monthly intervals for both factors, with no significant differences observed among cassava cultivars or plantlet sources at all sampling times (Figures 1(c), 1(d)). However, the HB60 and RY9 cultivars generally had higher canopy heights compared to the KU50 cultivar. Similarly, the total leaf number showed significant variation across cultivars, but no significant differences were observed between plantlet sources (Figures 1(e), 1(f)). Notably, the HB60 cultivar consistently exhibited a higher leaf number than the other cultivars. Specifically, from 4 MAP to 8 MAP, the leaf number of HB60 was 70, 85, 109, 117, and 161, respectively. In comparison, the KU50 cultivar had leaf numbers of 66, 63, 76, 71, and 90, while the RY9 cultivar had leaf numbers of 55, 62, 74, 69, and 85, respectively. Regarding plantlet sources, the average leaf number per plant ranged from 63 to 111 leaves between 4 MAP and 8 MAP.

Significant differences in internode length were observed among cassava cultivars, with the KU50 and RY9 cultivars having longer internodes than the HB60 cultivar, especially between 5 and 8 MAP. However, no significant differences were found between plantlet sources for internode length (Figures 1(g), 1(h)). Unlike other growth parameters, the number of branches showed significant differences for both cultivar and plantlet sources at all sampling times (Figures 1(i), 1(j)). The KU50 and HB60 cultivars exhibited a higher number of branches compared to the RY9 cultivar. Additionally, plants from leaf bud cuttings, mini-cuttings, and normal cuttings demonstrated a higher number of branches than the control group. However, this parameter displayed a high standard error due to the variability in branch development, with some plants showing branches and others not.

### 3.3. Growth and Development of Cassava Plantlets in a Field Environment

**Figure 2:**
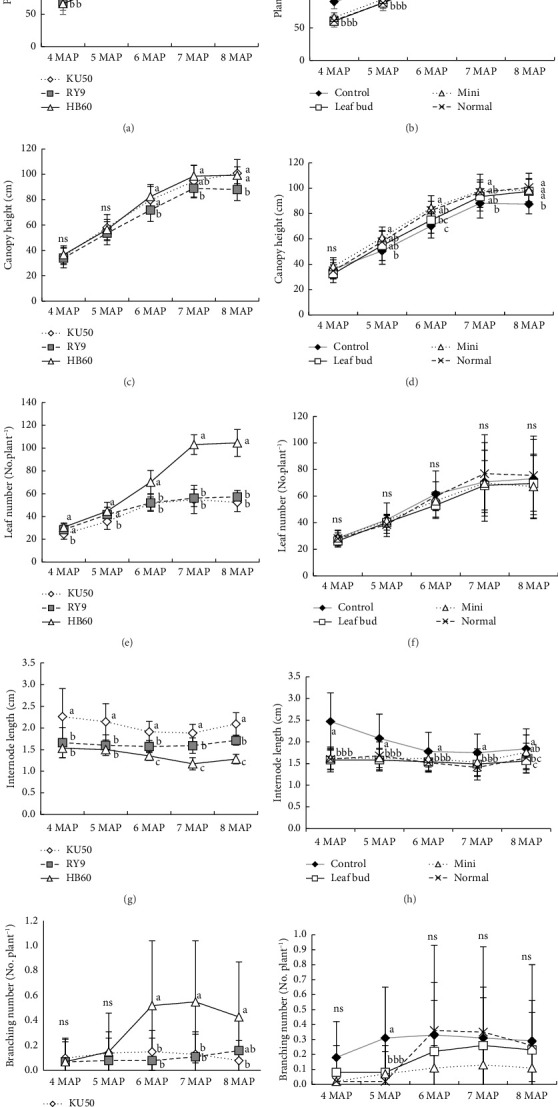
(a) & (b) plant height, (c) & (d) canopy height, (e) & (f) total leaf number, (g) & (h) internode length, and (i) & (j) branching number of three cassava cultivars and in four sources of plantlets under field experiment conditions. There was no significant interaction between cassava plantlet sources and cassava cultivars for all parameters. Different lowercase letters represent significant differences among cassava cultivars and cassava plantlets at *p-*value ≤ 0.05.

The field experiment results demonstrated statistically significant differences in plant height among cassava cultivars and plantlet sources at all sampling times (Figures 2(a), 2(b)). The KU50 cultivar exhibited greater height at 4-5 MAP compared to other cultivars, while at 6–7 MAP, both KU50 and HB60 exhibited significantly greater plant heights than the RY9 cultivar. By 8 MAP, however, the plant heights of HB60 and RY9 ranged from 190 to 197 cm, surpassing that of KU50, which reached 180 cm (Figure 2(a)).

Regarding plantlet sources, conventional cuttings consistently produced taller plants compared to leaf bud, mini-, and normal cuttings across all sampling times (Figure 2(b)). Similarly, canopy height showed significant differences for both factors across most sampling times (Figures 2(c), 2(d)). At 6-8 MAP, the KU50 and HB60 cultivars demonstrated significantly greater canopy heights than the RY9 cultivar (Figure 2(c)). Among plantlet sources, mini-cuttings achieved the greatest canopy height, followed by normal cuttings and leaf bud cuttings, during 5–8 MAP. Conversely, the control treatment resulted in the shortest canopy height compared to the treated plantlets (Figure 2(d)).

Remarkably, significant differences among cassava cultivars were observed for both leaf number and internode length (Figures 2(e), 2(g)). The HB60 cultivar recorded the highest leaf numbers, especially at 6–8 MAP, with values of 70, 103, and 105 leaves, respectively. In contrast, the KU50 cultivar exhibited the longest internodes, followed by RY9 and HB60. Among plantlet sources, significant differences in internode length were observed at nearly all sampling times. However, no significant differences in leaf number were recorded among plantlet sources (Figures 2(g), 2(h)). The plantlets derived from conventional cuttings produced the longest internodes (Figure 2(h)).

Branching patterns varied significantly among cultivars only at 6–8 MAP, with the HB60 cultivar producing more branches than the others. For plantlet sources, significant differences in branch number were observed only at 5 MAP, where control plants produced the highest number of branches (Figures 2(i), 2(j)).

### 3.4. Responses of Leaf Area, SLA, Multiplication Rate, and Stem Diameter Under Pot and Field Conditions

Cassava cultivars showed significant differences in most growth traits at the final harvest (8 MAP) in both pot and field experiments. In the pot experiment, notable traits included leaf area and stem diameter, while in the field experiment, leaf area, SLA, multiplication rate, and stem diameter showed significant variations (Table 2). The source of plantlets also affected certain traits, including stem diameter and multiplication rate in the pot experiment, as well as leaf area and stem diameter in the field experiment. Notably, there was no significant interaction between cassava cultivars and plantlet sources for most traits under both experimental conditions.

Among the cultivars, HB60 exhibited the largest leaf area in both experiments, with 29,727 cm^2^ per plant in the pot experiment and 27,520 cm^2^ per plant in the field experiment. Stem diameter also varied significantly among cultivars. In the pot experiment, HB60 (22.40 cm) and RY9 (22.67 cm) displayed greater stem diameters than KU50 (18.96 cm), while in the field experiment, HB60 (24.93 cm) demonstrated the largest stem diameter. Although SLA and multiplication rate did not differ significantly among cultivars in the pot experiment, significant differences were noted in the field experiment. HB60 showed the highest SLA (211.63), whereas KU50 and HB60 achieved the highest multiplication rates (4.61 and 4.25 per plant, respectively) in the field experiment.

The source of plantlets significantly impacted multiplication rate in the pot experiment, with control cuttings yielding the highest rate (7.75 per plant). A similar trend was observed in the field experiment, where control cuttings achieved the highest multiplication rate (4.48 per plant), although this was not significantly different from other plantlet sources. Stem diameter was also influenced by plantlet sources, with mini-cuttings, normal cuttings, and leaf bud cuttings producing the largest diameters in both pot and field experiments (Table 2).

### 3.5. Responses of Photosynthetic Traits at Different Growth Stages Under Pot and Field Conditions

Photosynthetic traits, including net photosynthetic rate (Pn), stomatal conductance (Gs), transpiration rate (Tr), intercellular CO_2_ concentration (Ci), maximum quantum yield efficiency of photosystem II in the light (Fv′/Fm′), and TE, were assessed at 4 and 6 MAP. In the pot experiment, significant variations among cassava cultivars were observed for Pn, Gs, and Tr at 4 MAP, while no significant differences were detected for Ci, Fv′/Fm′, or TE (Table 3). At 6 MAP, cassava genotypes exhibited significant differences in Gs, Tr, and Ci under pot conditions (Table 4).

The cultivars RY9 and HB60 showed the highest values for Pn (17.28 and 15.92 μmol CO_2_ m^−2^ s^−1^), Gs (0.46 and 0.40 mol H_2_O m^−2^ s^−1^), and Tr (9.40 and 9.20 mol H_2_O m^−2^ s^−1^) at 4 MAP in the pot experiment, outperforming the KU50 cultivar. At 6 MAP, RY9 also showed superior performance, with the highest recorded Gs (0.71 mol H_2_O m^−2^ s^−1^), Tr (8.84 mol H_2_O m^−2^ s^−1^), and Ci (304.75 μmol CO_2_ mol^−1^).

In contrast, no significant differences among cassava cultivars and plantlet sources were observed for any photosynthetic parameters (Pn, Gs, Tr, Ci, Fv′/Fm′, and TE) during field experiments or across plantlet sources in both pot and field experiments at either sampling time. Moreover, interactions between cultivars and plantlet sources were not significant for most photosynthetic traits, with the exception of Pn in the field experiment at 6 MAP (Table 4).

### 3.6. Responses of Biomass, Yields, Starch Content, and HI Under Pot and Field Conditions

In the pot experiment, significant differences among cassava cultivars were observed for almost all measured parameters, including the number of tubers per plant, tuber dry weight, biomass, starch content, starch yield, and HI, except for tuber fresh weight (Table 5). Notably, the RY9 and HB60 cultivars produced the highest values for several traits, including the number of tubers per plant (16.06 and 15.93 tuber plant^−1^), tuber dry weight (1.28 and 1.27 kg plant^−1^), biomass (1.68 and 1.80 kg plant^−1^), and starch yield (0.81 and 0.84 kg plant^−1^). HB60 recorded the highest starch content (26.25%), whereas KU50 and RY9 showed the highest HI values (0.75 and 0.76, respectively).

The source of plantlets under pot conditions significantly affected starch content and HI but did not affect the number of tubers per plant, tuber fresh weight, tuber dry weight, biomass, or starch yield. Leaf bud plantlets exhibited the highest starch content (26.08%), although the difference was not statistically significant compared to mini-cuttings (25.35%) and normal cuttings (24.83%). Similarly, leaf bud, mini-cutting, and normal cutting plantlets demonstrated higher HI values (0.74–0.75) than the conventional control (0.71). No significant interaction effects were detected between cultivars and plantlet sources for most traits, except for the number of tubers (Table 5).

In the field experiment, cultivar significantly affected most yield traits, except for starch yield. The RY9 and HB60 cultivars outperformed KU50 in key parameters, producing more tubers per plant (13.93 and 11.97 tubers plant^−1^), higher tuber fresh weight (34.45 and 35.84 tons ha^−1^), greater tuber dry weight (13.35 and 13.61 tons ha^−1^), higher biomass (17.80 and 19.63 tons ha^−1^), and greater starch content (25.20% and 25.53%). Additionally, RY9 and HB60 showed higher starch yields (8.70 and 8.55 tons ha^−1^) than KU50 (7.75 tons ha^−1^). Similar to the pot experiment, the field experiment revealed that KU50 and RY9 cultivars had higher HI values (0.73) than HB60 (0.69) (Table 5).

Plantlet sources in the field experiment significantly affected tuber fresh weight, tuber dry weight, starch yield, and HI, while other traits remained unaffected (Table 5). Leaf bud, mini-cutting, and normal cutting plantlets exhibited superior performance in significant traits, achieving tuber fresh weights of 32.05–36.56 tons ha^−1^, tuber dry weights of 12.16–13.96 tons ha^−1^, starch yields of 7.83–9.01 tons ha^−1^, and HI values of 0.73–0.76. Similar to the pot experiment, no significant interactions between cassava cultivars and plantlet sources were observed for most parameters, except for the number of tubers (Table 5).

## 4. Discussions

### 4.1. Differential Growth Responses of Cassava Cultivars and Plantlet Sources

In the pot experiment, no significant difference was observed among cassava cultivars for plant height and canopy height. However, significant variations were noted under field conditions (Figures 1(a), 1(b), 1(c), 1(d), 2(a), 2(b), 2(c), 2(d)). This discrepancy is likely due to the influence of a wider range of environmental variables in the field, such as variations in soil fertility, rainfall, and soil moisture, which were controlled in the pot experiment. Previous studies have demonstrated that factors such as soil fertility, planting dates, and soil water availability can significantly affect cassava growth patterns and yields [22, 23]. In our study, the KU50 cultivar exhibited significantly greater plant height from 4 to 7 MAP compared to RY9 and HB60 (Figure 2(a)). By 8 MAP, RY9 and HB60 had surpassed KU50 in height, likely due to their rapid late-stage growth, resulting in higher average plant and root weights [24]. Conventional cuttings exhibited greater plant height at most stages (4–7 MAP), but differences among cuttings were not significant by 8 MAP (Figure 2(b)). This aligns with Conceicão et al. [25], who reported that mini-cuttings (8 cm) resulted in similar growth to standard cuttings (16 cm) by 8 MAP.

KU50 and HB60 exhibited greater canopy heights, with significant differences from 6 to 8 MAP (Figure 2(c)). Canopy traits, such as leaf number, leaf area index (LAI), and stay-green ability, are crucial for cassava yield, as they influence light interception and photosynthesis [26, 27]. KU50's higher LAI during storage root accumulation supports its increased biomass and yield [28, 29]. The similar performance of KU50 and HB60 is likely due to their genetic relatedness, as HB60 was developed from a cross between RY5 and KU50 [30].

For the source of plantlets, those derived from plantlet sources revealed a greater canopy height than those from conventional cuttings (Figure 2(d)). Moun et al. [17] noted early leaf development in plantlets, contributing to their superior growth. The application of foliar fertilizers during early growth may have further enhanced plantlet performance.

Under pot conditions, KU50 and RY9 exhibited longer internodes than HB60 from 6 to 8 MAP (Figure 1(g)). In the field, KU50 showed the greatest internode length, followed by RY9 and HB60 from 6 to 8 MAP (Figure 2(g)). The shorter internode of HB60 may result from its greater leaf production, which diverts resources away from stem elongation. This aligns with observations of HB60's higher leaf count (Figures 1(e), 2(e)). The relationship between leaf production and internode length can be understood through the concept of resource allocation in plants. When a plant produces more leaves, it reallocates resources such as nutrients and energy to support leaf development. This shift in resource allocation often results in fewer resources being available for stem elongation, leading to shorter internodes [31]. HB60 cultivar demonstrated not only a higher number of leaves but also higher LA under both pot and field conditions compared to other cultivars, such as KU50 and RY9 (Table 2).

A previous study noted that the HB60 cultivar performed well under drought conditions, particularly during high storage root accumulation [32]. Generally, cassava cultivars with forking and branching growth patterns exhibit higher LAI and biomass compared to nonforking types [33]. Our study found that HB60, characterized as a forking and branching cultivar, consistently exhibited the highest number of leaves, branching numbers, and leaf area from 4 to 8 MAP for both pot and field experiments (Figures 1(e) and 2(e)). Leaves play a critical role in cassava growth by capturing sunlight for photosynthesis [34]. Numerous studies have shown strong correlations between LA, LAI, biomass accumulation, and yield [35, 36]. SLA is a key physiological trait, particularly in the context of drought tolerance, as it reflects the surface area available for photosynthesis relative to leaf biomass [37]. Notably, the HB60 cultivar had a higher SLA compared to the KU50 and RY9 cultivars (Table 2). SLA is positively correlated with leaf nitrogen content and Pn [38, 39], with higher SLA and nitrogen content generally contributing to enhanced Pn and Gs [40].

Under field conditions, normal cuttings exhibited significantly higher LA compared to leaf bud cuttings, mini-cuttings, and conventional cuttings (Table 2). However, this difference was not observed in the performance of plantlets grown under the pot experiment. Moun et al. [17] reported that normal cuttings demonstrated superior growth during the seedling stage, as evidenced by greater plantlet height, leaf number, root number, and plantlet diameter compared to leaf bud and mini-cuttings propagated aeroponically. Furthermore, positive correlations between plant height, leaf number, and key stem and root traits were recorded [41]. Growth parameters, including plant height, stem diameter, and leaf number, were strongly associated with final tuber yield, especially during the peak vegetative phase [42].

The number of branches increased significantly in plantlets derived from plantlet sources compared to conventional cuttings under the pot experiment (Figure 1(i)). However, no significant differences among plantlet sources were observed in the field experiment (Figure 2(i)). Similarly, Rusdi et al. [15] reported that normal cuttings outperformed mini-cuttings in branching and tuber yield for certain varieties. However, previous studies have highlighted the potential of mini-cuttings as a cost-effective and space-efficient alternative that may not significantly compromise yield. However, a high standard deviation (SD) was observed for this trait in both pot and field experiments. These variations likely contributed to the wider range of values, even within the same varieties and plantlet sources.

The stem diameter was significantly greater in both the RY9 and HB60 cultivars under pot conditions (Table 2). However, under field conditions, HB60 had a significantly greater stem diameter compared to RY9 and KU50 (Table 2). This enhanced stem diameter in HB60 may be attributed to its larger leaf area, observed in both pot (29,727 cm^2^ plant^−1^) and field experiments (27,520 cm^2^ plant^−1^). A previous study with six cassava varieties found a significant positive correlation between the number of leaves per plant and stem diameter [43]. Larger leaves demand greater structural support, which likely contributes to increased stem diameter [44]. Interestingly, the stem diameter of conventional cuttings (control) was significantly smaller than that of plantlet sources (Table 2). This difference could be explained by variations in their initial growth conditions. Conventional cuttings, derived from mature stems, may have been subjected to environmental stress or nutrient depletion, while plantlets are typically grown under controlled conditions with optimal nutrient management. These controlled conditions likely support more vigorous early growth, resulting in thicker stems. The quality of cuttings is generally influenced by various factors, including fertilizer application, planting density, and variety [45].

There were no significant differences among cassava cultivars in multiplication rates in the pot experiment, which ranged from 6.46 to 6.81 cuttings. However, a significant difference was observed in the field experiment (Table 2), with KU50 and HB60 showing the highest values. Under pot conditions, conventional cuttings (control) showed a significantly higher multiplication rate (7.75 cuttings) than other treatments (6.00–6.45 cuttings). This difference, however, was not observed under field conditions (Table 2). The treatments applied to the plantlets did not significantly affect their propagation success for the next growing season. Optimizing the use of these plantlets is important, as increasing the multiplication ratio of cassava stem cuttings is essential to addressing the shortage of propagation material [46].

### 4.2. Photosynthetic Trait Responses of Cassava Cultivars and Plantlet Sources

Our pot experiment demonstrated significant differences in photosynthetic parameters among cassava cultivars at 4 MAP. Notably, cultivars RY9 and HB60 consistently outperformed KU50 in terms of Pn, Gs, and Tr. Specifically, RY9 (17.28 μmol CO_2_ m^−2^ s^−1^) and HB60 (15.92 μmol CO_2_ m^−2^ s^−1^) exhibited higher Pn values compared to KU50 (12.33 μmol CO_2_ m^−2^ s^−1^) (Table 3). However, these Pn values were slightly lower than those reported in previous studies, which ranged from 20 to 35 μmol CO_2_ m^−2^ s^−1^ under optimal conditions [47]. In contrast to our findings, where RY9 exhibited higher Pn than KU50, a previous study reported no significant difference between these cultivars (15.81 and 16.59 μmol CO_2_ m^−2^ s^−1^, respectively) [26]. This discrepancy may be attributed to variations in planting seasons and environmental conditions, particularly soil characteristics. Our experiment was conducted in low-fertility, acidic soil, which may contribute to the lower Pn values compared to previous studies and the differential responses of the cassava cultivars. Consistent with earlier findings, Gago et al. [48] reported a positive correlation between Pn, Tr, and Gs. Gs serves as a key diffusional control, regulating CO_2_ entry for photosynthesis and water vapor loss through transpiration. By optimizing water loss and maintaining sufficient water availability, stomata ensure efficient photosynthetic activity [49]. In our study, RY9 and HB60 revealed similar Ci to KU50 (Table 3), yet their higher Pn/Ci ratios suggest greater efficiency in utilizing available CO_2_ for photosynthesis [50]. Cassava's exceptional sink strength, driven by efficient source–sink relationships, prevents the photosynthetic downregulation often observed in other crops under elevated CO_2_ conditions [51, 52]. This attribute enhances photosynthesis, water use efficiency, and yield under high CO_2_ conditions [36, 53]. At 6 MAP, similar trends were observed, with significant differences among cultivars in Gs, Tr, and Ci (Table 4). The consistency of the cultivar factor across time points underscores its importance in determining gas exchange parameters. RY9 exhibited the highest Gs values, indicating a superior capacity for gas exchange and potentially greater CO_2_ uptake for photosynthesis. However, this advantage in gas exchange did not translate into significantly higher Pn compared to KU50 and HB60. Interestingly, our findings diverge from those of Santanoo et al. [54], who reported higher Gs values for KU50 compared to RY9.

Our experiment revealed that Fv′/Fm′ values among cassava cultivars at 4 and 6 DAP ranged from 0.483 to 0.541, while among plantlet sources ranged from 0.502 to 0.544 across both pot and field experiments. No significant differences were observed among cultivars and plantlet sources for all sampling ages in both pot and field trials in terms of Fv′/Fm′ (Tables 3 and 4). These findings align with those of Sawatraksa et al. [55], who reported Fv′/Fm′ values of 0.51–0.53 for field-grown cassava at 4 MAP. This parameter reflects the maximum efficiency of Photosystem II in converting light energy for electron transfer to the acceptor plastoquinone (QA), and it varies with light intensity. It provides a nondestructive method for assessing the functional integrity of Photosystem II and serves as a crucial tool for understanding how crop varieties respond to different growing environments, and is used as one indicator to determine photosynthetic performance [56, 57]. This study presented that all cassava cultivars and plantlet sources have similar potential efficiency for Photosystem II reaction centers in capturing light energy for electron transport. The values in the pot-based system were lower compared to the field environment. By 6 MAP, the constrained soil volume likely led to significant root restriction and progressive nutrient depletion. Santanoo et al. [58] also reported that irrigated field cassava showed Fv′/Fm′ of approximately 0.53, which varied with climatic factors in each season. Our Fv′/Fm′ results were consistent with gas exchange parameters, which showed a similar pattern between Fv′/Fm′ and Pn (Tables 3 and 4).

Our study revealed no significant differences in TE among cassava cultivars, with consistent results across all sampling times in both pot and field experiments. This contrasts with previous studies under drought conditions. For instance, Wongnoi et al. [32] highlighted the importance of TE in water-limited environments. The lack of variations in TE among cultivars suggests that, under our experimental conditions, all tested cultivars performed similarly because they did not experience drought stress. Other factors, rather than cultivar type, appeared to have a greater influence on TE. Similarly, no significant differences among plantlet sources were observed for photosynthetic traits (Pn, Gs, Tr, Fv′/Fm′, and TE) in both pot and field experiments across all sampling times (Tables 3 and 4). All plantlet sources exhibited similar photosynthetic performance to conventional cuttings. The application of foliar fertilizer may have been a key factor in enhancing plantlet growth and maintaining plant health. Niu et al. [59] noted that foliar fertilization, when combined with soil fertilization, can effectively improve crop yield and enhance soil quality. Further research into the specific effects of foliar fertilization on plantlet development in our study could offer valuable insights for optimizing nursery practices. The positive correlation between growth parameters and yield can be attributed to increased photosynthetic capacity and assimilate production [60, 61].

### 4.3. Differential Yield Responses of Cassava Cultivars and Plantlet Sources

Cassava yield and biomass can vary significantly among different varieties [62, 63]. In our study, tuber fresh weight did not show significant differences among cultivars under pot conditions. However, in field conditions, HB60 (35.84 ton ha^−1^) and RY9 (34.45 ton ha^−1^) produced significantly higher tuber fresh weight than KU50 (30.38 ton ha^−1^) (Table 5). In the field experiment, HB60 and RY9 also exhibited superior growth traits, including plant height, leaf number, and stem diameter, which contributed to higher biomass, number of tubers, starch content, and tuber yields (both fresh and dry weights). These findings were consistent with the pot experiment, where RY9 and HB60 outperformed KU50 in terms of the number of tubers, tuber dry weight, starch content, and starch yield (Table 5). Our findings align with those of Malik et al. [64], who reported that HB60 had the highest yield among other varieties, although they did not compare it to KU50. The satisfactory performance of RY9 and HB60 in terms of yield may be attributed to their higher rates of Pn, Gs, and Tr at 4 MAP compared to KU50 (Table 3), which could have contributed to their greater yield. Pn, Gs, and Tr are crucial factors influencing cassava productivity [36]. Additionally, studies by Prasitsarn et al. [65], Vichukit et al. [66], and Wongnoi et al. [32] noted that RY9 and HB60 exhibited higher starch content than KU50. Yield differences among cassava cultivars can also be attributed to various factors, such as genetic potential, climatic conditions, soil types, and management practices, leading to performance variations across different locations. The lack of significant differences in the pot experiment may be due to the controlled environment, which could have limited the expression of genetic potential in terms of yield.

The plantlets derived from normal cuttings surprisingly exhibited higher tuber fresh weight than those from conventional cuttings, although this increase was not statistically significant compared to yields from plantlets derived from leaf bud or mini-cuttings, both of which were similar to conventional cuttings (Table 5). Notably, plantlets from normal cuttings exhibited a greater leaf area than those from other cutting types (Table 2), which may have contributed to the observed higher yield. An increased LAI and longer leaf lifespan are related to increased biomass production and stable yields, even under stressful conditions [67]. Domestication has led to increased SLA and Pn in cassava compared to its wild relatives [68]. In terms of overall yield, all plantlet sources performed similarly and exhibited comparable canopy height (Figure 2(d)). Canopy characteristics, such as leaf number, LAI, and stay-green ability, are known to positively correlate with yield [28].

Our findings implied that the highest starch content under pot conditions was observed in leaf bud plantlets (26.08%), compared to conventional cuttings (24.55%). However, this difference was not statistically significant when compared to mini-cuttings (25.35%) or normal cuttings (24.83%). Under field conditions, no significant differences in starch content were found among the various plantlet sources (Table 5). Starch content can vary depending on environmental factors [68–70]. In our study, plantlets were propagated in a greenhouse for 40 days before transplanting [17]. It is possible that the greenhouse conditions prior to transplanting contributed to the higher starch accumulation observed in the plantlets.

However, the plantlet source did not significantly affect yield performance in terms of the number of tubers, total fresh weight, tuber dry weight, biomass, and starch yield in the pot experiment. Under field conditions, the number of tubers, starch content, and biomass were similarly unaffected by plantlet source. Nonetheless, significant differences were observed for other traits: plantlets derived from leaf bud, mini-cutting, and normal cutting methods produced significantly higher starch content and HI than conventional cuttings in the pot experiment. These plantlet sources also yielded significantly higher tuber fresh weight, tuber dry weight, starch yield, and HI compared to conventional cuttings in the field experiment (Table 5). The discrepancies between pot and field experiments may be attributed to several key factors, primarily environmental conditions and differences in root development. Field conditions present a more complex and variable environment compared to the controlled conditions of pot experiments. This variability in the field may have allowed for the expression of differences between plantlet sources that were not observable in the pot environment. Root development plays a crucial role in these differences. The restricted space in pots likely limited root growth, potentially masking differences between plantlet sources. In contrast, field conditions provide unrestricted space for root growth, leading to more pronounced differences in plant performance [71]. The open soil environment in the field enables roots to explore a larger volume of soil, potentially accessing more nutrients and water, thereby revealing performance differences that were not observable in the pot experiment.

These factors, either individually or in combination, offer a plausible explanation for why certain yield components showed significant differences in the field experiment but not in the pot experiment. Importantly, plantlets derived from leaf bud, mini-cutting, and normal cuttings significantly outperformed conventional cuttings in several yield traits under field conditions. Similarly, in the pot experiment, these alternative methods showed superior performance for some traits, although differences for other traits were not significant compared to conventional cuttings. The superior performance of plantlets from leaf buds, mini-cuttings, and normal cuttings can be attributed to their early growth under controlled greenhouse conditions, where they received optimal nutrient management. These conditions likely promoted more vigorous early growth, enhanced photosynthetic traits, and larger stem sizes, leading to higher yields later in the growing season, as evidenced by our results.

## 5. Conclusion

This study investigated the growth, yield, and photosynthetic traits of three cassava cultivars and four plantlet sources under pot and field conditions. The results indicated that HB60 and RY9 were the highest yielding cultivars, exhibiting superior growth characteristics, including a greater number of leaves, higher leaf area with thicker leaves (high SLA), wider stem diameter, and enhanced photosynthetic parameters (Pn, Gs, and Tr). These cultivars also produced higher yields. RY9 showed strong potential for rapid propagation, demonstrating increased plant height, stem diameter, and a high multiplication rate. The plantlet sources did not significantly impact photosynthetic traits under either pot or field conditions. Interestingly, leaf bud, mini-cutting, and normal cutting plantlets outperformed other sources regarding key traits, such as tuber fresh weight, tuber dry weight, starch yield, and HI. No significant interaction was observed between cassava cultivars and plantlet sources for most parameters. This indicates that the performance of leaf bud cuttings, mini-cuttings, and normal cuttings was comparable to conventional planting methods in terms of photosynthetic traits, yield, and HI. Furthermore, the similar responses of cassava cultivars to various plantlet sources indicate that these plantlet sources could be a valuable consideration for plant propagation programs.

## Figures and Tables

**Table 1:** The physical and chemical properties of the soil analyzed prior to transplanting in both pot and field experiments.

Properties	Pot experiment	Field experiment	
		0–30 cm	30–60 cm
pH (1:1)	5.65	5.04	4.98
Electrical conductivity (1:5) (ds m^−1^)	0.02	0.01	0.01
Organic matter (%)	0.57	0.48	0.25
Total nitrogen (%)	0.04	0.02	0.01
Available phosphorus (mg kg^−1^)	100.63	13.75	5.13
Exchangeable potassium (mg kg^−1^)	24.16	6.84	6.69
Exchangeable calcium (mg kg^−1^)	113.71	22.99	10.63
Exchangeable magnesium (mg kg^−1^)	29.39	9.31	4.60
Cation exchange capacity (cmol kg^−1^)	10.70	4.20	5.00
Sand (%)	77.87	75.92	74.95
Silt (%)	19.19	20.95	20.76
Clay (%)	2.94	3.13	4.29
Texture	Loamy sand		

*Note:* pH (1:1): pH meter, electrical conductivity (1:5): electrical conductivity meter, organic matter: Walkley and Black method, available phosphorus: Bray II, spectrophotometer (molybdenum blue), exchangeable potassium and exchangeable magnesium: ammonium acetate leaching, atomic absorption spectrophotometry, cation exchange capacity: ammonium saturation, distillation, soil texture: mechanical analysis with the pipette method.

**Table 2:** Growth traits of cassava cultivars derived from various plantlet sources in the pot and field experiments.

Factors	Pot experiment				Field experiment			
	Leaf area (cm^2^ plant^−1^)	SLA (cm^2^ g^−1^)	Multiply rate (number plant^−1^)	Stem diameter (cm)	Leaf area (LA) (cm^2^ plant^−1^)	SLA (cm^2^ g^−1^)	Multiply rate (number plant^−1^)	Stem diameter (cm)
Cultivar (*C*)								
KU50	17,952^b^	205.39	6.81	18.96^b^	18,567^b^	199.39^b^	4.61^a^	21.21^c^
RY9	19,739^b^	203.36	6.46	22.40^a^	18,612^b^	199.76^b^	4.08^b^	22.81^b^
HB60	29,727^a^	214.01	6.46	22.67^a^	27,520^a^	211.63^a^	4.25^ab^	24.93^a^

F-test	^∗∗^	ns	ns	^∗∗^	^∗∗^	^∗∗^	^∗^	^∗∗^

Sources (*S*)								
Control	21,037	207.35	7.75^a^	20.35^b^	19,615^b^	201.48	4.48	21.70^b^
Leaf bud	21,038	204.88	6.00^b^	21.56^ab^	21,305^b^	204.50	4.22	23.24^ab^
Mini	24,242	210.18	6.45^b^	22.45^a^	20,763^b^	203.91	4.44	23.52^a^
Normal	23,573	207.93	6.12^b^	21.02^ab^	24,582^a^	201.48	4.11	23.48^a^

F-test	ns	ns	^∗∗^	^∗^	^∗∗^	ns	ns	^∗^

*C* × *S*	ns	ns	^∗^	ns	^∗∗^	ns	ns	ns

C.V. (%)	22.78	7.22	19.82	8.49	11.05	4.12	9.38	5.27

*Note:* Different lowercase letters represent significant differences among cassava cultivars and cassava plantlets at *p-*value ≤ 0.05.

Abbreviations: C.V. = coefficient of variation, ns = nonsignificant, SLA = specific leaf area.

^∗^Significant at *p* ≤ 0.05.

^∗∗^Significant at *p* ≤ 0.01.

**Table 3:** Photosynthetic traits of cassava cultivars derived from various plantlet sources in the pot and field experiments at 4 months after planting.

Factors	Pot experiment						Field experiment					
	Pn	Gs	Tr	Ci	Fv′/Fm′	TE	Pn	Gs	Tr	Ci	Fv′/Fm′	TE
Cultivar (*C*)												
KU50	12.33^b^	0.27^b^	7.80^b^	287.19	0.509	1.64	22.13	1.69	9.37	315.50	0.498	2.39
RY9	17.28^a^	0.46^a^	9.40^a^	291.75	0.512	1.85	22.17	1.66	9.53	318.17	0.483	2.36
HB60	15.92^a^	0.40^a^	9.00^a^	289.94	0.506	1.79	22.85	1.69	9.89	318.50	0.517	2.31

F-test	^∗∗^	^∗∗^	^∗∗^	ns	ns	ns	ns	ns	ns	ns	ns	ns

Sources (*S*)												
Control	14.42	0.35	8.11	290.50	0.509	1.86	22.84	1.27	9.65	309.33	0.498	2.38
Leaf bud	15.85	0.39	9.24	287.75	0.506	1.71	21.67	1.64	9.61	321.67	0.495	2.26
Mini	15.08	0.38	8.73	290.83	0.502	1.74	23.19	1.85	9.90	317.11	0.509	2.36
Normal	15.36	0.39	8.83	289.42	0.520	1.73	21.84	1.96	9.23	321.44	0.496	2.40

F-test	ns	ns	ns	ns	ns	ns	ns	ns	ns	ns	ns	ns

*C* × *S*	ns	ns	ns	ns	ns	ns	ns	ns	ns	ns	ns	ns

C.V. (%)	17.03	31.58	12.45	4.25	5.90	20.39	7.24	41.83	8.02	3.56	8.24	8.71

*Note*: Pn = net photosynthesis (μmol CO_2_ m^−2^s^−1^), Gs = stomatal conductance (mol H_2_O m^−2^s^−1^), Tr = transpiration rate (mol H_2_O m^−2^s^−1^), Ci = intercellular carbon dioxide concentration (μmol CO_2_ mol^−1^), Fv′/Fm′ = maximum quantum yield efficiency of Photosystem II in the light, TE = transpiration efficiency (μmol CO_2_ mol^−1 ^H_2_O). Different lowercase letters represent significant differences among cassava cultivars and cassava plantlets at *p-*value ≤ 0.05.

Abbreviations: C.V. = coefficient of variation, ns = nonsignificant.

^∗∗^Significant at *p* ≤ 0.01.

**Table 4:** Photosynthetic traits of cassava cultivars derived from various plantlet sources in the pot and field experiments at 6 months after planting.

Factors	Pot experiment						Field experiment					
	Pn	Gs	Tr	Ci	Fv′/Fm′	TE	Pn	Gs	Tr	Ci	Fv′/Fm′	TE
Cultivar (*C*)												
KU50	19.33	0.55^b^	8.61^ab^	294.63^ab^	0.531	2.37	20.14	0.96	10.49	304.58	0.541	1.97
RY9	19.84	0.71^a^	8.84^a^	304.75^a^	0.519	2.37	20.90	1.26	11.81	312.08	0.528	1.78
HB60	17.87	0.43^b^	7.76^b^	281.71^b^	0.532	2.39	20.74	1.27	10.97	309.75	0.533	1.94

F-test	ns	^∗∗^	^∗∗^	^∗∗^	ns	ns	ns	ns	ns	ns	ns	ns

Sources (*S*)												
Control	19.64	0.62	8.23	294.64	0.540	2.51	20.33	1.09	11.19	305.56	0.533	1.89
Leaf bud	18.72	0.59	8.52	296.33	0.529	2.31	20.49	1.01	11.08	304.11	0.544	1.87
Mini	18.65	0.52	8.23	291.67	0.522	2.42	20.76	1.38	11.09	313.33	0.532	1.91
Normal	19.04	0.53	8.64	292.14	0.518	2.27	20.81	1.17	11.01	312.22	0.528	1.93

F-test	ns	ns	ns	ns	ns	ns	ns	ns	ns	ns	ns	ns

*C* × *S*	ns	ns	ns	ns	ns	ns	^∗^	ns	ns	ns	ns	ns

C.V. (%)	12.05	30.15	11.13	5.37	4.18	14.43	5.52	42.67	11.88	5.61	3.08	13.23

*Note*: Pn = net photosynthesis (μmol CO_2_ m^−2^s^−1^), Gs = stomatal conductance (mol H_2_O m^−2^s^−1^), Tr = transpiration rate (mol H_2_O m^−2^s^−1^), Ci = intercellular carbon dioxide concentration (μmol CO_2_ mol^−1^), Fv′/Fm′ = maximum quantum yield efficiency of Photosystem II in the light, TE = transpiration efficiency (μmol CO_2_ mol^−1 ^H_2_O). Different lowercase letters represent significant differences among cassava cultivars and cassava plantlets at *p-*value ≤ 0.05.

Abbreviations: C.V. = coefficient of variation, ns = nonsignificant.

^∗∗^significant at *p* ≤ 0.01.

**Table 5:** Yield traits of cassava cultivars derived from various plantlet sources in the pot and field experiments.

Factors	Pot experiment							Field experiment						
	NT	TFW	TDW	Biomass	SC	SY	HI	NT	TFW	TDW	Biomass	SC	SY	HI
Cultivar (*C*)														
KU50	13.78^b^	2.82	1.03^b^	1.38^b^	24.43^b^	0.68^b^	0.75^a^	10.26^b^	30.38^b^	11.81^b^	15.99^b^	23.71^b^	7.75	0.73^a^
RY9	15.93^a^	3.28	1.28^a^	1.68^a^	24.93^b^	0.81^a^	0.76^a^	13.93^a^	34.45^ab^	13.35^ab^	17.80^ab^	25.20^a^	8.70	0.73^a^
HB60	16.06^a^	3.15	1.27^a^	1.80^a^	26.25^a^	0.84^a^	0.70^b^	11.97^ab^	35.84^a^	13.61^a^	19.63^a^	25.53^a^	8.55	0.69^b^

F-test	^∗∗^	ns	^∗∗^	^∗^	^∗∗^	^∗∗^	^∗∗^	^∗∗^	^∗^	^∗^	^∗∗^	^∗^	ns	^∗∗^

Sources (*S*)														
Control	16.00	3.08	1.13	1.59	24.55^b^	0.75	0.71^b^	11.03	30.22^b^	11.73^b^	17.17	24.90	7.57^b^	0.68^b^
Leaf bud	14.45	2.99	1.22	1.63	26.08^a^	0.78	0.75^a^	12.31	32.05^ab^	12.16^ab^	16.62	24.45	7.83^ab^	0.73^a^
Mini	15.00	3.09	1.22	1.65	25.35^ab^	0.81	0.74^ab^	11.98	35.40^ab^	13.85^a^	18.34	25.43	9.01^a^	0.76^a^
Normal	15.58	3.18	1.20	1.61	24.83^ab^	0.79	0.75^a^	12.89	36.56^a^	13.96^a^	19.10	24.49	8.29^ab^	0.73^a^

F-test	ns	ns	ns	ns	^∗^	ns	^∗∗^	ns	^∗^	^∗^	ns	ns	^∗^	^∗∗^

C × S	^∗^	ns	ns	ns	ns	ns	ns	^∗^	ns	ns	ns	ns	ns	ns

C.V. (%)	12.06	18.81	17.47	17.83	4.69	18.02	3.66	16.46	12.65	12.93	11.22	4.59	12.66	3.70

*Note*: NT = number of tubers plant^−1^, TFW = tuber fresh weight plant^−1^ (Pot = kg plant^−1^, Field = ton ha^−1^), TDW = tuber dry weight plant^−1^ (Pot = kg plant^−1^, Field = ton ha^−1^), Biomass (Pot = kg plant^−1^, Field = ton ha^−1^), SC = starch content (%), SY = starch yield (Pot = kg plant^−1^, Field = ton ha^−1^). Different lowercase letters represent significant differences among cassava cultivars and cassava plantlets at *p-*value ≤ 0.05.

Abbreviations: C.V. = coefficient of variation, HI = harvest index, ns = nonsignificant.

^∗^significant at *p* ≤ 0.05.

^∗∗^ significant at *p* ≤ 0.01.

## Data Availability

The data that support the findings of this study are available in the supporting information of this article.
